# PDL1 shapes the classical Hodgkin lymphoma microenvironment without inducing T-cell exhaustion

**DOI:** 10.3324/haematol.2022.280014

**Published:** 2022-07-14

**Authors:** Joseph G. Taylor, Edward Truelove, Andrew Clear, Maria Calaminici, John G. Gribben

**Affiliations:** 1 Centre for Haemato-Oncology, Barts Cancer Institute, Queen Mary University of London; 2 Barts NHS Trust, St Bartholomew’s Hospital, London, UK

## Abstract

Classical Hodgkin lymphoma (CHL) is unusually sensitive to PD1 inhibition and PDL1 is highly expressed on CHL cells and in the tumor microenvironment. This could be interpreted as evidence of exhaustion, but paradoxically, PD1^+^ lymphocyte infiltration does not predict response to PD1 inhibitors and no increase in cytotoxic markers is seen after PD1 therapy as might be expected with reversal of exhaustion. In contrast to PD1, elevated PDL1 does predict response to PD1 inhibitors and recent data associate both retained CHL MHC-II expression and increased T helper (T_H_) T-cell receptor diversity with response, suggesting a connection to the T_H_ compartment. We performed a phenotypic, spatial and functional assessment of T-cell exhaustion in CHL and found co-expression of an exhaustion marker and lower PD1 expression in CHL than in reactive nodes whereas the proliferative and cytokine production capacity were similar in CHL and the reactive nodes. We found no correlation between PDL1 expression and exhaustion signatures. Instead, we identified a strong association between PDL1 expression and CHL MHC-II expression, T_H_ recruitment, and enrichment of T_H_1 regulatory cells. These data suggest that a dominant effect of PDL1 expression in CHL may be T_H_ engagement and promotion of a regulatory microenvironment rather than maintenance of exhaustion.

## Introduction

Classical Hodgkin lymphoma (CHL) is the only licensed indication for CD279 (PD1, programmed cell death protein 1) inhibitors in hematologic malignancies and is unusually sensitive to this therapy.^1,2^ PD1 inhibitors are classically thought to act by reversing T-cell exhaustion, a state which limits the effectiveness of anti-tumor immune responses.^3^ Effector T cells become exhausted when they are chronically stimulated by low levels of antigen.^4^ Exhausted cells have sustained high expression of PD1, alongside other exhaustion markers, including CD223 (LAG3), TIM3, TBC21 (TBET) and EOMES, and progressively lose their effector functions.^4,5^ Exhaustion is partially reversible and PD1 inhibition can reinvigorate the T-cell response leading to improved tumor clearance.

CHL cells express high levels of ligand 1 for PD1 (PDL1) and polysomy, copy gains and amplifications of the *PDL1* locus are seen in upwards of 95% of cases.^6,7^ PDL1 expression is also prominent within other cells in the tumor microenvironment.^8^ However, functional data demonstrating exhaustion in CHL are limited and most studies, including studies from our own laboratory, report only low PD1 expression in the CHL microenvironment.^9^ Furthermore, PD1^+^ cell infiltration does not predict response to PD1 inhibitors in CHL.^9,10^ Recent studies demonstrate that during PD1 inhibitor therapy the CHL microenvironment is characterized by a rapid reduction in PDL1^+^ macrophages and type 1 regulatory cells (T_R_1) rather than cytotoxic T-cell expansion that might be expected with reversal of T-cell exhaustion.^11^ Another study found that expansion of singleton (putatively newly immigrant) T helper (T_H_) clones is associated with PD1 inhibitor response.^12^ This is in line with data from solid tumors and suggests that during PD1 therapy activated tumor-specific T cells are in fact newly immigrant and not derived from exhausted populations that were present before therapy.^13^ These studies run counter to the traditional model of PD1 inhibitors acting by reversing exhaustion and highlight a need to better understand the function of PDL1 within the CHL microenvironment.

In this study we phenotypically and functionally assessed exhaustion in CHL and its relationship to PDL1 expression. Given the evidence of a close connection between PD1 inhibition and T_H_ cells, we further assessed the relationship between PDL1 and T_H_ effector subsets in CHL. We found low levels of exhaustion and no evidence of a connection between an exhausted phenotype and PDL1 expression. However, we identified a strong connection between PDL1 expression and enrichment for the T_H_1_Reg_ regulatory phenotype, a recently characterized subset of T regulatory cells expressing both FOXP3 and TBET which may specialize in suppression of T_H_1 cells that are numerous in the CHL microenvironment. These data support and expand upon recent findings that PDL1 expression in CHL may have a closer relationship to the maintenance of a protective tumor microenvironment than to the mediation of exhaustion.

## Methods

Human tissue was accessed as part of study 05/Q0605/140, approved by the Regional Ethics Committee, with consent through Barts Tissue Bank and National Health Service Blood and Transplant. Cell lines were obtained from DSMZ. Samples were identified by a systematic search of adult cases at St Bartholomew’s Hospital between 1999 and 2016. The diagnosis was confirmed in all cases by an expert hematopathologist (MC) and Epstein-Barr virus (EBV) status was determined by EBV-encoded small RNA *in situ* hybridization (sample characteristics in Table 1). Reactive lymph nodes (RLN) from adult patients were selected as controls. Cases positive for human immunodeficiency virus or associated with malignancy were excluded. RLN histology was follicular hyperplasia (64%), progressive transformation of germinal centers (8%) or not otherwise specified (28%). RLN provide a comparative non-malignant immune response within lymphoid tissue and have been used in previous studies.^9^ Non-reactive nodal tissue is scarce, usually collected during staging of known malignancy and generally small with bland infiltrate and so was considered a less robust control.

### Immunohistochemistry

Immunohistochemistry (IHC) and multiplex IHC studies were performed on tissue microarrays. Forty-seven CHL cases were randomly selected for the multiplex experiments and grouped on a single array. Further details are provided in the *Online Supplementary Methods*.

**Table 1.**
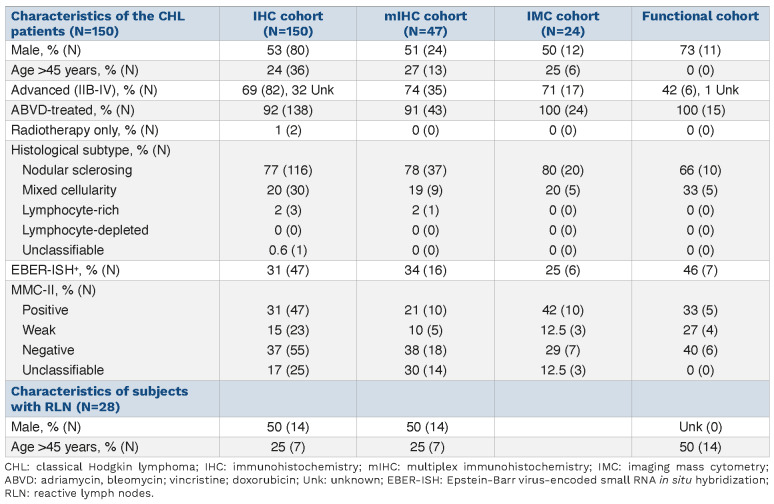
Characteristics of the cohort of patients.

### Imaging mass cytometry

Imaging mass cytometry was performed on tissue micro-arrays on a subset of 24 cases from the multiplex IHC array. Further details are provided in the *Online Supplementary Methods*.

### Cell culture and flow cytometry

For assessment of macrophage PDL1, KMH2-conditioned medium was harvested from high-viability, low-passage cells after 24 hours at 1x10^6^/mL in RPMI medium. Monocytes from healthy donor peripheral blood mononuclear cells were positively selected using anti-CD14 microbeads according to the manufacturer’s instructions before being seeded in 2 mL RPMI medium at 5x10^5^/mL in six-well plates with macrophage colony-stimulating factor 50 ng/mL and granulocyte-macrophage colony-stimulating factor 1 ng/mL. On day 7, macrophages were treated with RPMI or 50% RPMI/conditioned medium. Cells were harvested at 24 hours for flow cytometry analysis. For exhaustion assays, single cell suspensions were stimulated in 200 mL RPMI medium at 1x10^6^/mL in 96-well plates. For cytokine and Ki67 assays, cells were stimulated with phorbol myristate acetate/ionomycin with protein transport inhibitors for 4 hours at 37°C. For proliferation assays the cells were stimulated with 10 mg/mL anti-CD3 and 1 mg/mL anti-CD28 and incubated for 4 days at 37°C after staining with CFSE. For co-cultures, healthy donor naïve CD4^+^ T cells were separated from peripheral blood mononuclear cells with the Stemcell EasySep kit according to the manufacturer’s instructions before co-culture at 1x10^6^/mL with 4x10^4^/mL KMH2 for 8 days before analysis by flow cytometry. Data were acquired using a BD LSRFortessa with compensation and fluorescence minus one controls at every run. The data were then analyzed using FlowJo software. Further details are available in the *Online Supplementary Methods*.

### Analyses

Images were analyzed in Visiopharm and R. Package details are provided in the *Online Supplementary Methods*. Spatial distributions were compared to the parent cell distribution (e.g., CD8^+^ to CD3^+^ cells) to avoid confounding data due to distribution of parent cell-type or complete spatial randomness where no biologically appropriate parent cell comparator was available. Tukey style boxplot marking was used to show medians and interquartile ranges.

## Results

### Classical Hodgkin lymphoma cells are PDL1^hi^ and induce a PDL1^+^ myeloid microenvironment

**Figure 1.**
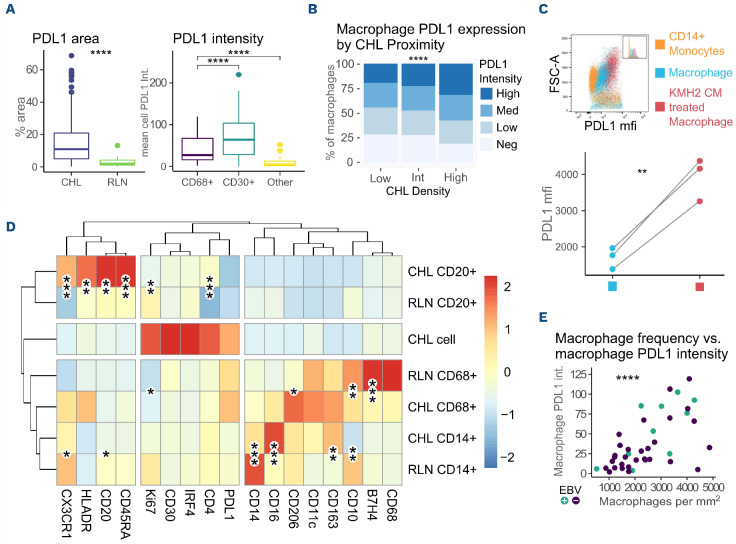
**Classical Hodgkin lymphoma cells express high levels of PDL1 and exist within a PDL1-positive microenvironment.** (A) Classical Hodgkin lymphoma (CHL) is associated with increased PDL1 expression with highest expression intensity on CHL cells. Left: total PDL1 area by immunohistochemistry of 149 cases of CHL (EBV+ 46, EBV- 103) and 15 reactive lymph node (RLN). Right: mean cell PDL1 intensity by multiplex immunohistochemistry (IHC) in CD30+ (CHL cells), CD68+ (macrophages) and CD30-CD68-(other) cells in cases of CHL (n=46). (B) Macrophage PDL1 intensity increases with CHL density. Relative frequency of PDL1^High^, PDL1^Med^, PDL1^Low^ and PDL1^Neg^ macrophages within tessellation areas of CHL tumor. Tessellation areas were calculated by CHL cell density. The c2 test was used for statistical comparisons, *P*<0.0001, mean across 47 cases. (C) Monocyte-derived macrophages upregulate PDL1 in the presence of media conditioned by the KMH2 CHL cell line. Healthy donor CD14+ monocyte-derived macrophages cultured with (red) or without (blue) KMH2 conditioned media for 24 h. Flow cytometry analysis was performed on three biological replicates. mfi: mean fluorescence intensity. (D) Phenotyping of microenvironmental cells in CHL in comparison to RLN. The mean cell expression averaged by case of CD20+ (B cells), CD68+ (macrophages), CD14+ (monocytes) and CHL cells in CHL or RLN by imaging mass cytometry. Asterisks denote statistical significance of the comparison of mean cell intensity by case (20 CHL, 10 RLN). (E) Macrophage PDL1 intensity correlates with macrophage frequency. Mean PDL1 intensity per CD68+ cell compared to CD68+ cell frequency was assessed by multiplex immunohistochemistry in 47 CHL cases (EBV+ 9, EBV- 38), R=0.61, (EBV+ R= 0.72, EBV- R=0.55). Comparisons were performed with Mann-Whitney and Wilcoxon rank (three-way) tests. Correlations were performed by Pearson rank. ns: non-significant, *P<0.05, **P<0.01, ***P<0.001, ****P<0.0001.

We quantified PDL1 expression by IHC in 150 patients with CHL and in 28 age-matched patients with RLN. The characteristics of the cohorts are summarized in Table 1. PDL1 expression on CHL cells (CD30^+^) and macrophages (CD68^+^) was further assessed by multiplex IHC in 47 CHL cases on a single tissue microarray. PDL1 expression was elevated in CHL (with expression seen on both CHL cells and within the microenvironment) (*P*<0.0001) (Figure 1A). This effect was seen irrespective of EBV status (*data not shown*). Mean PDL1 intensity was higher in CHL cells than in macrophages (*P*<0.0001) (Figure 1A). CD68^+^ macrophage frequency was increased in regions of high CHL density (*P*<0.0001). PDL1 intensity increased with macrophage proximity and density to CHL cells (*P*<0.0001) (Figure 1B). Consistent with this, media conditioned by the KMH2 CHL cell line induced up-regulation of PDL1 on monocyte-derived macrophages *in vitro* (*P*<0.01) (Figure 1C). PDL1 upregulation in myeloid cells co-cultured with CHL cell lines is consistent with published data.^14^ Media conditioned with the non-Hodgkin lymphoma cell lines DHL4 and DHL6 also induced PDL1 expression on monocyte-derived macrophages, but at significantly lower levels than the CHL cell line (*data not shown*). Phenotyping of microenvironmental antigen-presenting cells (CD68^+^ macrophages, CD20^+^ B cells and CD14^+^ monocytes) by imaging mass cytometry and comparison to RLN demonstrated elevated CD206 intensity but lower B7H4 intensity (both M2 markers) in CHL than CD68^+^ in RLN. PDL1 expression was highest in CHL cells and CD68^+^ macrophages. CD14^+^ monocytes had higher CD16 and CD163 expression but lower mean CD14 expression than their RLN counterparts whereas CD20^+^ B cells were more likely to express HLADR, CX3CR1 and CD45RA (Figure 1D).

Together, these data confirm, as previously reported, that CHL cells both express high levels of PDL1 and exist in a macrophage-enriched, PDL1^+^ microenvironment.^8,15^ Further to this, our data demonstrate that microenvironmental PDL1 expression is mainly seen within the CD68^+^ macro-phage compartment, and that there is a correlation between macrophage number and the intensity of macrophage PDL1 expression (R=0.6, *P*<0.0001) (Figure 1E), suggesting that tumors promoting macrophage recruitment were also those promoting microenvironmental PDL1 expression.

### PD1^+^ and phenotypically exhausted cells are infrequent in classical Hodgkin lymphoma, despite high PDL1 expression

**Figure 2.**
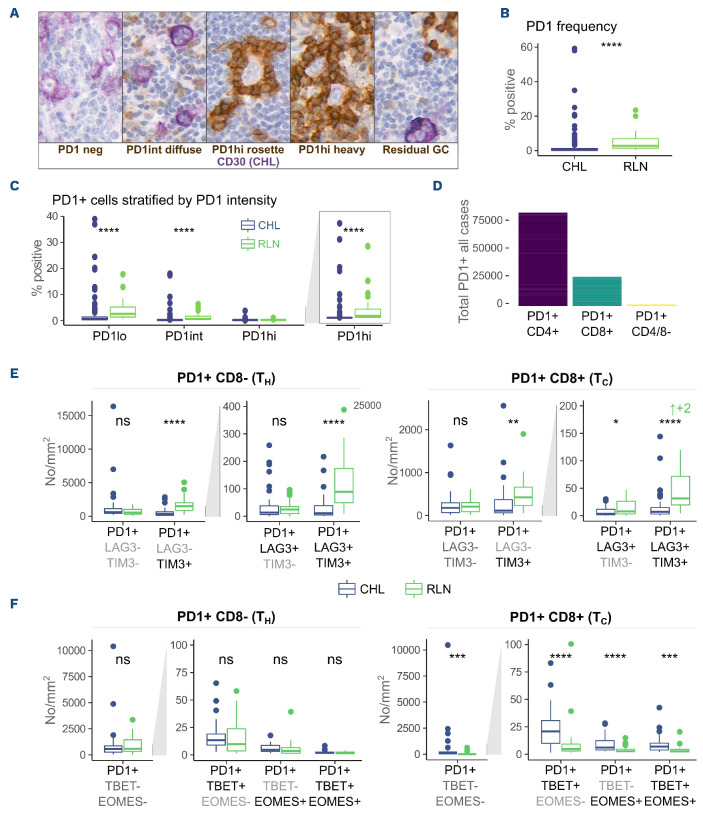
**PD1-positive lymphocytes are less frequent in classical Hodgkin lymphoma than in reactive lymph nodes.** (A) Multiple PD1 infiltration patterns were observed. Most cases were characterized by rare/low or diffuse PD1^weak/int^ infiltration. Rarely, PD1^hi^ cells rosetted around classical Hodgkin lymphoma (CHL) cells or heavy infiltration with PD1^hi^ cells was observed. Infrequent residual germinal centers were observed. Germinal centers were excluded from the analysis. In the images, PD1 is represented by brown, CD30 CHL by purple. (B) PD1^+^ infiltrating lymphocytes are reduced in CHL relative to reactive lymph node (RLN). Comparison by immunohistochemistry (IHC), germinal centers excluded (n=27/150). (C) PD1^+^ infiltrating lymphocytes are reduced in CHL irrespective of PD1 expression intensity. PD1^+^ area stratified by PD1 intensity by IHC. Germinal centers excluded. (right: PD1^hi^ cells on expanded axis) (n=27/150). (D) PD1^+^ expression in CHL is T-cell restricted with most expression in CD4^+^ cells. Left: PD1, CD4 and CD8 co-expression as determined by multiplex IHC. Given this, CD8 positivity was used to differentiate cytotoxic T cells (T_C_) from T helper cells (T_H_) in multiplex panels for identifying exhaustion. (E) PD1^+^ cells in CHL co-express LAG3 and TIM3 at lower frequency than PD1^+^ cells in RLN. PD1 co-expression with TIM3 and LAG3 was assessed in T_H_ and T_C_ by multiplex IHC. Validation demonstrated T-cell-restricted PD1 positivity. PD1^+^ T_H_ cells are defined as PD1^+^CD8^-^ and PD1^+^ T_C_ as PD1^+^CD8^+^. No significant difference was detected in PD1^+^ LAG3^–^TIM3^–^ T_H_ or T_C_ frequency between CHL and RLN. Cells co-expressing PD1 with LAG3, TIM3 or both were significantly less frequent in CHL than in RLN. PD1^+^LAG3^–^TIM3^–^ was the predominant T_H_ phenotype in CHL whereas PD1^+^TIM3^+^LAG3^–^ was predominant in CHL T_C_ and RLN T_H_. (F) PD1^+^ T_H_ cells co-express TBET and EOMES at similar frequency in CHL and RLN, while PD1^+^ T_C_ co-express these markers at a higher frequency. PD1, TBET and EOMES co-expression was assessed in the same cohort by multiplex IHC. No significant difference in PD1 co-expression with TBET or EOMES was identified within the T_H_ compartment. In contrast, increased co-expression was seen in the T_C_ compartment in CHL as compared to RLN. The predominant T_H_ and T_C_ phenotype was that of isolated PD1^+^ expression with co-expression rarely identified. Comparisons were made by the Wilcoxon Mann-Whitney test. ↑+2 indicates that two outliers were excluded from the graph. All outliers were included in the statistical analysis. ns: non-significant, *P<0.05, **P<0.01, ***P<0.001, ****P<0.0001.

After confirming that CHL cells and macrophages in the tumor microenvironment express high levels of PDL1 we sought evidence of a corresponding exhausted PD1^+^ T-cell population. Exhausted T cells are characterized by sustained PD1 expression and models suggest that cells with intermediate PD1 expression (PD1^int^) are most amenable to reversal by PD1 inhibition.^3,16^ We therefore quantified PD1 expression by IHC and stratified the results by PD1 expression intensity. Lymph node germinal centers (and rare residual germinal centers in 5/150 CHL cases) were excluded from analysis because of the high PD1 expression on T follicular helper cells (T_FH_). The analysis was performed using the most sensitive of three anti-PD1 monoclonal antibody clones tested and results were confirmed with a second clone (*Online Supplementary Figure S4*). PD1 expression was heterogeneous among cases, with patterns ranging from rare or no PD1 positivity to diffuse infiltration of PD1^int^, rosetting PD1^hi^ cells and rare cases of marked PD1^hi^ infiltration (Figure 2A). Overall, despite prominent CD3, CD4 and CD8 infiltration, PD1 expression was significantly less frequent in CHL than in RLN (Figure 2B). PD1^+^ cells were seen at lower frequency in CHL than in RLN irrespective of PD1 expression intensity (Figure 2C). No difference in PD1 expression was seen according to EBV status, but enrichment was seen in the lymphocyte-rich histological subtype, consistent with previous reports.^17^

Isolated PD1 expression is not specific for exhaustion.^18^ We therefore assessed co-expression with other markers of exhaustion by multiplex IHC whereby tissue was stained with a panel of IHC antibodies and then the IHC antibody was serially stripped off before re-staining with a new one. Stained images were virtually aligned allowing assessment of cell-marker co-expression. Multiplex IHC was chosen over imaging mass cytometry due to the larger cohort size and analysis of larger tissue areas (three tissue microarray cores *versus* 1x1 mm) making it preferable for rarer cell populations. PD1 expression was restricted to small lymphocytes and was expressed primarily on CD4^+^ cells, with some expression on CD8^+^ cells and minimal non-T-cell expression (Figure 2D). Since CD4 staining produced background when combined with TIM3 and LAG3 in stripping panels, after confirming PD1 T-cell restriction, CD4 was omitted from subsequent stripping panels. PD1^+^CD8^+^ and PD1^+^CD8^–^ lymphocytes were significantly less frequent in CHL than in RLN controls (Figure 2E). PD1^+^CD8^–^ lymphocytes co-expressed TBET or EOMES at similar levels to RLN controls while PD1^+^CD8^+^ lymphocytes co-expressed TBET and EOMES above levels in RLN (Figure 2F). Isolated PD1 expression was the dominant phenotype with very low levels of PD1/TBET/EOMES co-expression.

In summary, despite a more prominent T-cell infiltrate, PD1^+^ lymphocytes were less frequent in CHL than in RLN, irrespective of the intensity of PD1 expression and there was no enrichment of PD1^+^CD8^–^ lymphocytes co-expressing exhaustion markers in CHL. The dominant phenotype of both PD1^+^CD8^–^ and PD1^+^CD8^+^ lymphocytes in CHL was expression of PD1 in the absence of co-expression of any other exhaustion marker.

### Classical Hodgkin lymphoma T cells are proliferative, cytokine-competent and contain fewer functionally exhausted cells than do reactive lymph node T cells

Proliferation, interleukin 2 (IL2) and interferon gamma (IFNγ) production in T cells from single cell suspensions from diagnostic CHL lymph nodes compared to five RLN single cell suspensions were assessed to determine functional features of T_H_ and cytotoxic T (T_C_) exhaustion.^3–5^ Paired formalin-fixed, paraffin-embedded samples were evaluated to ensure that the single cell suspension samples were representative of the IHC cohort. No significant difference in PDL1, CD68, PD1, TBET, GATA3 or RORγT expression was seen, but FOXP3 expression was marginally higher in the single cell suspension group of samples than in the wider IHC cohort (*Online Supplementary Figure S5*). The characteristics of the cohorts are described in Table 1.

**Figure 3.**
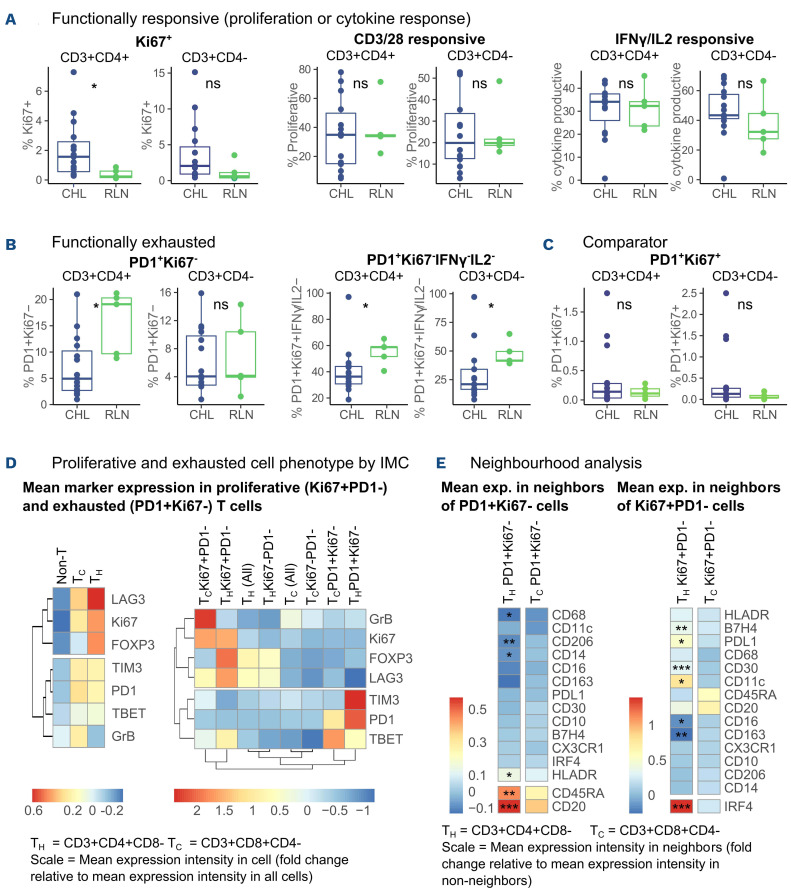
**Classical Hodgkin lymphoma T cells are proliferative, cytokine-competent and contain fewer functionally exhausted cells than T cells from reactive lymph nodes.** (A) Classical Hodgkin lymphoma (CHL) T cells are proliferative and cytokine-competent. Left: unstimulated T_H_ cells from CHL single cell suspensions expressed higher percentages of Ki67 than T_H_ from reactive lymph node (RLN) single cell suspensions (*P*=0.017). Middle: CHL T cells were no less proliferative than RLN T cells to CD3/CD28 stimulation, as measured by CFSE assay after 3 days of culture *in vitro*. Right: CHL T cells produced no less cytokines than RLN T cells in response to phorbol myristate acetate (PMA)/ionomycin stimulation, as measured by intracellular flow cytometry after 4 h of PMA/ionomycin stimulation *in vitro*. (B) Functionally exhausted cells are less frequent in CHL. Left: non-proliferative (Ki67^–^) T_H_ cells were less frequent in CHL (unstimulated, *P*=0.019). Right: non-proliferative non-cytokine productive (Ki67^–^IFNγ/IL2^–^) T_C_ and T_H_ cells were less frequent in CHL, as measured by intracellular flow cytometry after 4 h of PMA/ionomycin stimulation *in vitro* (*P*=0.025 and *P*=0.015, respectively). CHL, n=15; RLN, n=5. (C) Comparator cells. Indeterminate (both Ki67^+^ and PD1^+^) cells provided as a comparison group to proliferative and functionally exhausted groups (unstimulated, *P*=ns). CHL, n=15; RLN, n=5. Comparisons performed using the Mann-Whitney test. (D) Proliferative and exhausted cell phenotypes as determined by imaging mass cytometry. Mean per-cell marker expression intensity in the cell of interest was expressed as fold change from mean value across all cells. Results were calculated by sample and the overall mean results are displayed. Left: expression intensities in non-T (all CD3^–^), T_C_ (CD3^+^CD8^+^CD4^–^) or T_H_ (CD3^+^CD4^+^CD8^–^) cells. Right: expression intensities in proliferative and exhausted T-cell subgroups. (E) Neighborhood analysis of mean marker expression in cells adjacent to proliferative or exhausted T-cell subsets. Mean per-cell marker expression intensity in neighboring cells expressed as fold change from mean value across all cells. Results were calculated by sample and overall mean results are displayed. Comparisons were made by paired Mann-Whitney tests.

CHL suspensions had higher baseline Ki67^+^ T_H_ frequencies compared to the RLN controls. No significant differences in proliferation or IL2 and IFNγ production in response to stimulation were seen between CHL and RLN (Figure 3A). Ki67^–^PD1^+^ (non-proliferative PD1^+^) CD3^+^CD4^+^ cells were significantly less frequent in CHL than in RLN, while Ki67^–^PD1^+^ CD3^+^CD4^–^ cells were present at similar levels (Figure 3B). Non-cytokine-productive Ki67^–^PD1^+^ CD3^+^CD4^+^ and CD3^+^CD4^–^ cells were also significantly less frequent in CHL (Figure 3B). By comparison, no significant difference was seen in numbers of PD1^+^Ki67^+^ cells (Figure 3C). On subgroup analysis, CD3^+^CD4^–^ proliferation and CD3^+^CD4^+^ cytokine production capacity were significantly higher in EBV^+^ CHL than in EBV^–^ CHL (*Online Supplementary Figure S6*). Further phenotyping by imaging mass cytometry demonstrated elevated expression of TIM3 and modest elevation of TBET in the PD1^+^Ki67^–^(CD4^+^CD3^+^) T_H_ population, consistent with identification of an exhausted phenotype. In contrast, the proliferative Ki67^+^PD1^–^(CD8^+^CD3^+^) T_C_ expressed higher levels of granzyme B, while the Ki67^+^PD1^–^(CD4^+^CD3^+^) T_H_ fraction expressed increased FOXP3 and TBET. Interestingly LAG3 expression was higher in the T_H_ proliferative fraction, perhaps reflecting the presence of T_R_1 cells which are known to be enriched in CHL (Figure 3D).^19^ Analysis of mean marker expression in immediate neighbors of phenotypically exhausted T_H_ cells revealed higher expression of CD20, CD45RA (expressed on B cells in addition to naïve T cells) and HLADR with no relationship to CD30, PDL1 or IRF4 (CHL cell markers). In contrast, neighboring cells of phenotypic ally proliferative T_H_ cells expressed higher levels of IRF4, CD30, PDL1 and B7H4 (Figure 3E).

### PDL1 expression is not associated with PD1 expression in classical Hodgkin lymphoma

**Figure 4.**
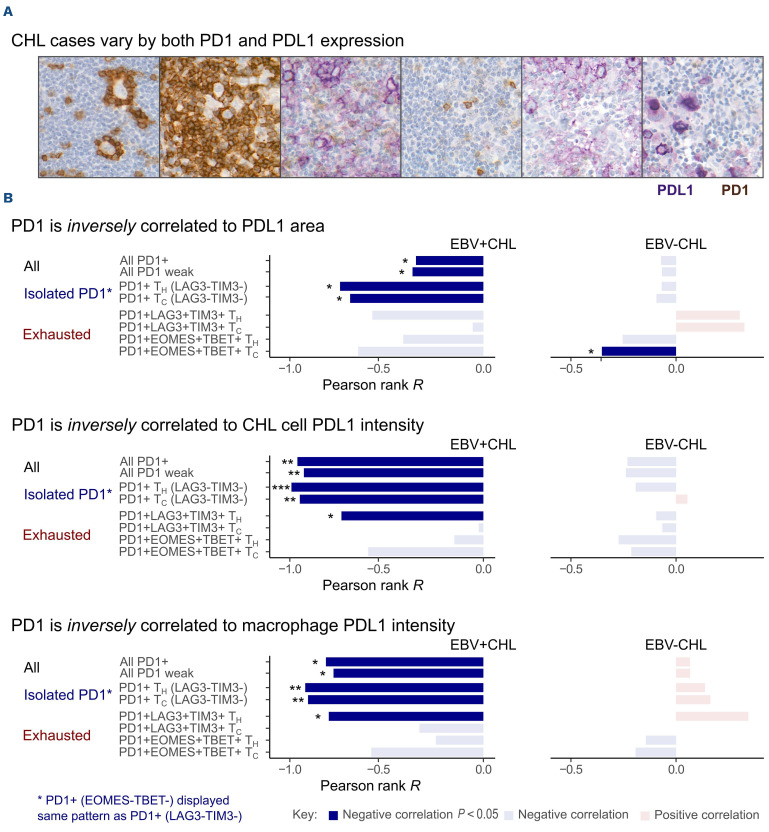
**PDL1 is not associated with markers of exhaustion.** (A) Cases vary by PD1 and PDL1 infiltration. Cases dominated by PD1^hi^ infiltration were consistently PDL1^–^, while PD1^weak^ and PDL1 cases varied so cases were observed with infiltration of one or both. (B) No correlation was seen between PD1 and PDL1 in classical Hodgkin lymphoma, whereas negative correlations to PDL1 area and intensity were seen in the Epstein-Barr virus (EBV)^+^ CHL subgroup. Negative associations were observed between PDL1 area, CHL PDL1 intensity and macrophage PDL1 intensity and PD1 in EBV^+^ CHL, with no significant correlation detected in the EBV^–^ group (EBV^+^ n=47, PD1^+^ *P*=0.03, PD1^wk^ *P*=0.02, EBV^–^ n=105. Pearson rank). Multiplex immunohistochemistry demonstrated that this effect was present in PD1^+^ cells not co-expressing exhaustion markers (LAG3/TIM3 or EOMES/TBET). PD1 expression in this context may represent an alternate state to exhaustion. No consistent association was seen in the EBV^–^ group (EBV^+^ n=10, EBV^–^ n=31, Pearson rank). The graph denotes strength of correlation and the dark color denotes statistical significance (*P*<0.05).

In the light of our findings of low PD1 expression and little functional evidence of exhaustion but high PDL1 expression we assessed the relationship between PD1 and PDL1 expression across CHL cases. Cases varied by both PDL1 and PD1 expression (Figure 4A), but counterintuitively, no association was detected between PD1 and total PDL1 area or PDL1 intensity in CHL, irrespective of stratification for PD1 intensity or analysis of exhaustion marker co-expression (Figure 4B). Unexpectedly, in EBV^+^ CHL an inverse relationship was observed between PD1 expression and PDL1 intensity (Figure 4B). Analysis of exhaustion marker co-expression demonstrated that isolated PD1 expression rather than co-expression of exhaustion markers (LAG3/TIM3 or EOMES/TBET) was responsible for this effect (Figure 4B). Therefore, compared to RLN controls, we detected significantly fewer phenotypic or functionally exhausted (non-cytokine productive, non-proliferative, PD1^+^) CD3^+^CD4^+^ (T_H_) and CD3^+^CD4^–^ (T_C_) cells and significantly more Ki67^+^ CD3^+^CD4^+^ (T_H_) cells in the CHL group, with no overall loss in proliferative or cytokine production capacity.

### PDL1 is associated with classical Hodgkin lymphoma MHC-II expression, T_H_ cell recruitment and local T_C_ exclusion

In the light of the above data, we considered alternative mechanisms by which PDL1 might influence the CHL microenvironment. We focused on its relationship to T_H_ populations given that both CHL cell MHC-II expression and CD4^+^ T-cell receptor diversity predict PD1 inhibitor response.^6,12^ This is in contrast to MHC-I, whose expression does not predict PD1 inhibitor response.^20^

**Figure 5.**
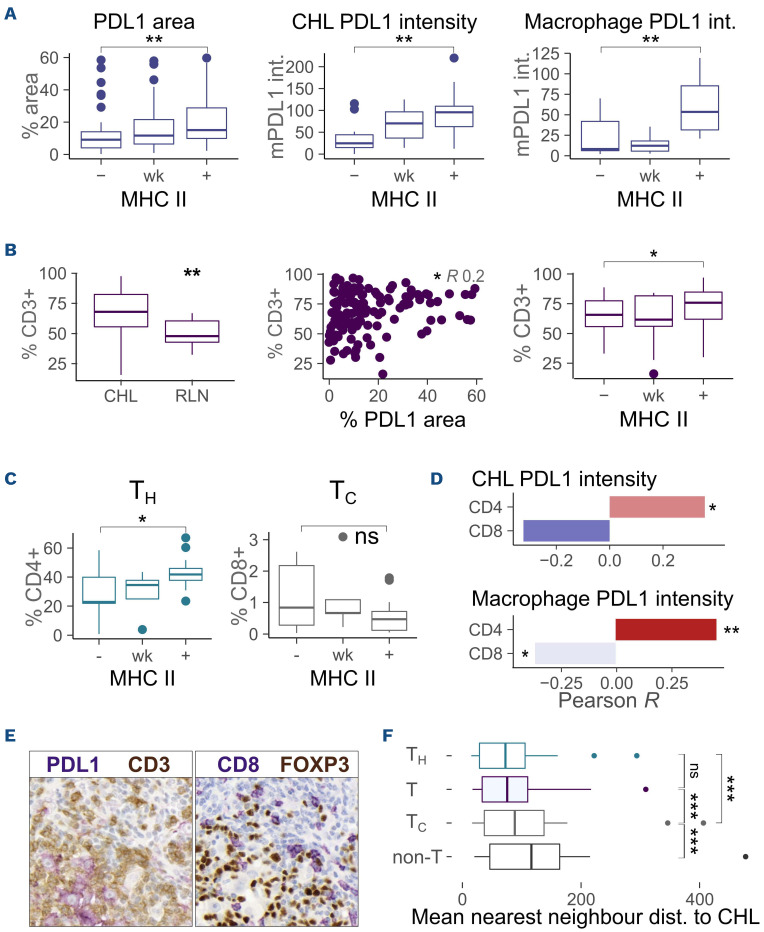
**PDL1 is associated with classical Hodgkin lymphoma MHC-II expression, T_H_ cell recruitment and local T_C_ cell exclusion.** (A) PDL1 area, mean classical Hodgkin lymphoma (CHL) PDL1 intensity and macrophage PDL1 intensity are positively associated with CHL major histocompatibility complex II (MHC-II) expression, as determined by multiplex immunohistochemistry (IHC). PDL1 area to MHC-II positive *versus* negative status, compared by a Mann-Whitney test. MHC-II manual scoring (n=47/23/55), CHL cell PDL1 intensity to MHC-II (n=11/6/17), macrophage PDL1 intensity to MHC-II (n=11/6/17). (B) CD3^+^ T cells are enriched in CHL and are associated with both PDL1 and CHL MHC-II expression, as determined by IHC. Left: n=145/13. Middle: R=0.2 (n=144). Right: MHC-II positive *versus* negative status (n=48/23/54). (C) CD4^+^ T_H_ but not CD8^+^ T_C_ infiltration is positively associated with CHL MHC-II expression. MHC-II positive *versus* negative status (n=32), *P*=0.022 and *P*=0.19, respectively. (D) CD4^+^ T_H_ infiltration in CHL is correlated with CHL cell and macrophage PDL1 intensity. Top: correlation by Pearson rank of CD4 and CD8 infiltration with CD30^+^ CHL cell PDL1 intensity, as determined by multiplex IHC. The graph denotes the strength of the correlation (R). Color intensity and stars denote statistical significance (n=32), (CD4 R=0.36). Bottom: CD4/CD8 to CD68^+^ macrophage PDL1 intensity (CD4 R=0.45, CD8 R= -0.39). (E) The image shows T-cell enrichment around PDL1^+^ tumor with local T_Reg_ enrichment and T_C_ exclusion. Left: CD3 (T) cells are stained brown and PDL1 (PDL1^+^ tumor) cells purple. Right: FOXP3 (T_Reg_) cells are stained brown and CD8 (T_C_) purple. (F) T_C_ are locally depleted and T_H_ enriched proximal to CHL cells. Mean nearest neighbor distance to CD30^+^IRF4^+^ CHL cells was assessed by imaging mass cytometry. T_H_ cells are CD3^+^CD4^+^CD8^–^, T_C_ cells are CD3^+^CD8^+^CD4^–^, T cells are CD3^+^, and non-T cells are CD3^–^ (CHL cases, n=20, Wilcoxon paired rank). MHC-II expression –: negative; wk: weak, +: positive; T_H_: T helper cells; T_C_: cytotoxic T cells. ns: non-significant, *P<0.05, **P<0.01, ***P<0.001, ****P<0.0001.

A positive association was observed between PDL1 expression and CHL MHC-II (Figure 5A), suggesting higher PDL1 expression in CHL cells retaining T_H_ antigen-presenting capacity or deriving other survival advantage from interaction with T_H_ cells in the tumor microenvironment. Of note, no association was identified between PD1 or exhaustion marker expression and CHL MHC II (*Online Supplementary Figure S7*). CD3 infiltration was increased in CHL and was positively associated with both PDL1 staining and CHL cell MHC II expression (Figure 5B). T_H_ cells were responsible for this pattern: CD4 infiltration correlated positively with PDL1 expression, CHL cell PDL1 intensity, CD68^+^ macrophage PDL1 intensity and CHL MHC-II expression, while a negative relationship was seen between these markers and CD8 infiltration (Figure 5C, D). Spatial data supported these observations. CD4^+^ and CD8^+^ cells were spatially segregated within the tumor, with CD4^+^ cells proximal to CHL cells while CD8^+^ cells were frequently excluded from the tumor microenvironment (Figure 5E). The image shows CD8 *versus* overall CD3 distribution with PDL1 identifying tumor. FOXP3 demonstrates local T_Reg_ enrichment). Consistent with this, (CD3^+^CD8^+^) T_C_ cells were located significantly further from CHL cells than both CD4^+^CD3^+^ T_H_ and CD3^+^ T cells overall in the imaging mass cytometry analysis (Figure 5F).

We therefore identified an association between PDL1, CHL MHC-II expression and CD4^+^ enrichment but CD8^+^ cell exclusion. Given that CHL cells secrete cytokines and actively recruit T_H_ cells this suggests that CHL cases with high PDL1 expression may be those that actively recruit and interact with T_H_ cells.^21^

### PDL1 and classical Hodgkin lymphoma MHC-II are associated with differentiation of T_H_ cells to T_H_1_Reg_ but not to T_H_1 or non-polarized T_Reg_ cells

**Figure 6.**
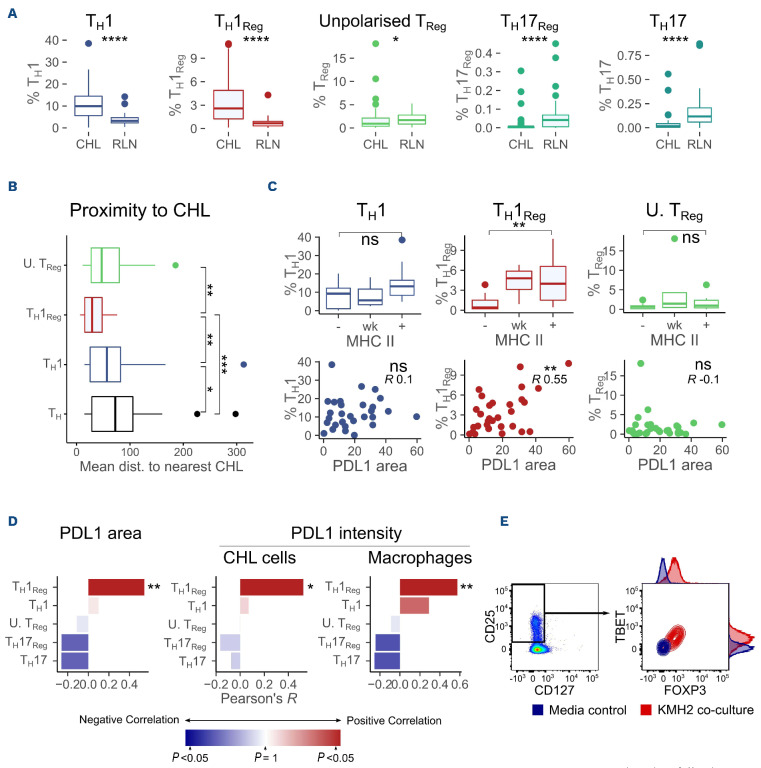
**PDL1 and classical Hodgkin lymphoma MHC-II expression are associated with skewed T_H_ differentiation towards T_H_1_Reg_ but not T_H_1 or non-polarized T_Reg_.** (A) T_H_1 and T_H_1_Reg_ are enriched in classical Hodgkin lymphoma (CHL) compared to reactive lymph nodes (RLN) while T_H_17, T_H_17_Reg_ and unpolarized T_Reg_ are depleted. TBET^+^FOXP3^+^CD4^+^ T_H_1_Reg_ (CD8^–^RORγT^–^) and TBET^+^CD4^+^ T_H_1 (FOXP3^–^RORγT^–^) were increased but FOXP3^+^CD4^+^ T_Reg_ (TBET^–^RORγT^–^), RORγT^+^CD4^+^T_H_17 (TBET^–^FOXP3^–^) and RORγT^+^FOXP3^+^CD4^+^ T_H_17_Reg_ (CD8^–^TBET^–^) were reduced, as determined by multiplex immunohistochemistry (IHC) (n=49/31). (B) T_H_1_Reg_ are located closer to CHL cells than either T_H_1 or non-polarized T_Reg_. Mean nearest neighbor distance to CD30^+^IRF4^+^ CHL cells for TBET^+^FOXP3^+^CD4^+^CD3^+^ T_H_1_Reg_, TBET^+^CD4^+^CD3^+^ T_H_1 (FOXP3^–^) and non-polarized FOXP3^+^CD4^+^CD3^+^ T_Reg_ (TBET^–^) measured relative to T_H_ (CD3^+^CD4^+^), as evaluated by imaging mass cytometry (n=20, CHL cases). (C) T_H_1_Reg_ but not T_H_1 or unpolarized (U) T_Reg_ are associated with both PDL1 and CHL MHC-II expression. There was increased TBET^+^FOXP3^+^CD4^+^ T_H_1_Reg_ (RORγT^–^) but not FOXP3^+^CD4^+^ T_Reg_ (TBET^–^RORγT^–^) or TBET^+^CD4^+^ T_H_1 (FOXP3^–^RORγT^–^) in CHL, as determined by multiplex IHC (MHC-II - to +; n=9/5/18). PDL1 area (n=32, T_H_1: R=0.1, T_H_1_Reg_: R=0.55, T_Reg_: R= -0.1). (D) T_H_1_Reg_ but not T_H_1 or non-polarized T_Reg_ correlate to PDL1 area and both macrophage and CHL cell mean PDL1 intensity. N=32, CD30^+^ CHL PDL1 intensity or CD68^+^ macrophage PDL1 intensity. (E) Co-culture of naïve T_H_ cells with the KMH2 CHL cell line promotes T_H_1_Reg_ differentiation. Naïve healthy donor T_H_ cells were co-cultured for 14 days with KMH2 cells or CD3/CD28 stimulation. KMH2 induced differentiation to CD3^+^CD4^+^CD25^hi^CD127^lo^FOXP3^+^TBET^+^ T_H_1_Reg_ relative to media-activated controls. T_H_1_Reg_ were PD1^hi^. Comparisons were made by the Mann-Whitney test, with spatial comparison by Wilcoxon paired rank and correlation by Pearson rank analysis. MHC-II expression –: negative; wk: weak, +: positive; T_H_: T helper cells. ns: non-significant, *P<0.05, **P<0.01, ***P<0.001, ****P<0.0001.

We next assessed the relationship between PDL1 and CHL MHC-II expression and T_H_ cell differentiation in CHL. Naïve T_H_ cells differentiate to effector subtypes with different functional roles based upon signals during activation. T_H_1 and T_H_17 cells are considered to have anti-tumor roles while T_H_2 and T_Reg_ are tumor protective. T_Reg_ also differentiate into subtypes mirroring effector types and may specialize in suppression of specific effector responses (T_H_1_Reg_ being more effective at suppressing a T_H_1 response).^22^ PD1 is upregulated on activation and PDL1-PD1 signaling modulates differentiation, driving development away from T_H_1 towards T_Reg_ subtypes.^23,24,24–27^ PDL1 also regulates the conversion of differentiated T_H_1 to T_Reg_ and stabilizes inducible T_Reg_ conversion to T_H_1_Reg_.^27–30^ PDL1 expression in MHC-II-positive CHL may therefore drive T_H_1_Reg_ differentiation to combat the anti-tumor T_H_1 response. To assess this, we first quantified the master transcription factors TBET, FOXP3, RORγT and GATA3 (for T_H_1, T_Reg_, T_H_17 and T_H_2, respectively) by conventional IHC. TBET^+^ and FOXP3^+^ cells were enriched in CHL while RORγT^+^ cells were reduced and no difference was detected in GATA3^+^ (*data not shown*). We then used a multiplex IHC panel including CD4, TBET, FOXP3, RORγT and CD8 to define T_H_1 (TBET^+^CD4^+^, FOXP3^–^RORγT^–^CD8^–^), T_H_1_Reg_ (TBET^+^FOXP3^+^CD4^+^, RORγT^-^CD8^-^), non-polarized T_Reg_ (FOXP3^+^CD4^+^, TBET^–^RORγT^–^ CD8^–^), T_H_17 (RORγT^+^CD4^+^, TBET^–^FOXP3^–^CD8^–^) and T_H_17_Reg_ (RORγT^+^FOXP3^+^CD4^+^, TBET^–^CD8^–^). T_H_1 and T_H_1_Reg_ were enriched in CHL, whereas T_H_17, T_H_17_Reg_ and, surprisingly, nonpolarized T_Reg_ were reduced (Figure 6A).

Next, we analyzed the spatial location of these T-cell subsets relative to CHL by imaging mass cytometry. T_H_1_Reg_ were located significantly closer to (CD30^+^IRF4^+^) CHL cells than both T_H_1 and non-polarized (TBET^–^) T_Reg_ (Figure 6B). CD30 and IRF4 were selected as both are immunohisto-chemical diagnostic markers of CHL. Given these findings we assessed the relationship between T_H_ polarization and both PDL1 and CHL MHC-II expression. Retained expression of MHC-II on CHL was associated with increased T_H_1_Reg_, but no association was seen with T_H_1 or non-polarized T_Reg_. Significant correlations were observed between T_H_1_Reg_ and PDL1 area, CHL PDL1 intensity and macrophage PDL1 intensity, but again no association was seen with T_H_1 or non-polarized T_Reg_ (Figure 6C, D). On subgroup analysis T_H_1_Reg_ associations with PDL1 retained significance in both EBV^+^ CHL and EBV^–^ CHL and in nodular sclerosing and mixed cellularity histological subtypes (*data not shown*). Associations with MHC-II expression were retained in EBV^–^ CHL and nodular sclerosing subgroups. MHC-II negative cases were too infrequent in EBV^+^ and mixed cellularity groups for statistical comparison. Finally, co-culture of naïve T_H_ from healthy donors with the KMH2 CHL cell line promoted marked T_H_1_Reg_ differentiation (CD3^+^CD4^+^CD25^hi^CD127^lo^FOXP3^+^TBET^+^) relative to media-activated controls. (Figure 6E) This is in line with previous publications.^31^ These data reveal that T_H_1_Reg_ (but not T_H_1 or non-polarized T_Reg_) enrichment is associated with high PDL1 and retained expression of MHC-II in CHL.

**Figure 7.**
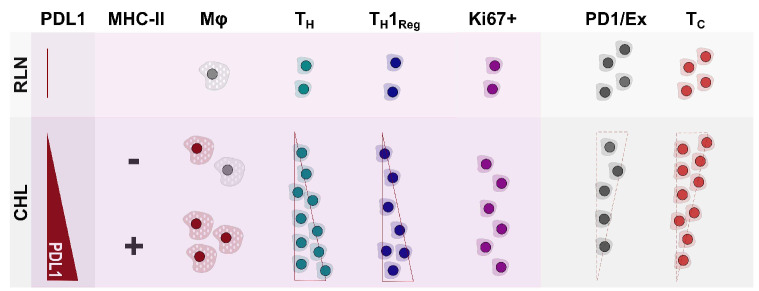
**Summary of key associations.** Comparison of classical Hodgkin lymphoma (CHL) cells to reactive lymph node cells shows a significant increase in PDL1 expression with enrichment of macrophages, T_H_, T_H_1_Reg_ and Ki67^+^ cells. PD1^+^/exhausted cells are seen at significantly lower frequency. Within CHL, there are positive associations between PDL1 and CHL cell MHC-II expression, macrophage PDL1 expression, and T_H_ and T_H_1_Reg_ infiltration whereas negative or absent associations are seen between PDL1 and both T_C_ and PD1^+^ or exhausted cell infiltration. CHL: classical Hodgkin lymphoma; RLN: reactive lymph nodes; PDL1: programmed death ligand 1; MHC-II major histocompatibility cell type II; Mφ: macrophages; T_H_: T helper cells; T_H_1_Reg_: T_H_1 regulatory cells; PD1/Ex: programmed death protein-1-positive/exhausted cells; T_C_: cytotoxic T cells.

Taken together these data show a lack of enrichment of PD1^+^ or exhausted cells in CHL and a negative or absent association between PD1 infiltration and PDL1 expression. Instead there is enrichment of Ki67^+^ cells and a positive association between PDL1 expression in CHL, retained MHC-II expression and both T_H_ and T_H_1_Reg_ cells (Figure 7).

## Discussion

We present data suggesting a role for PDL1 in shaping the CHL tumor microenvironment. We found similar levels of T-cell exhaustion in RLN controls and CHL and no relationship between exhaustion signatures and PDL1 expression. Instead, we identified a consistent association between PDL1 and T_H_ recruitment, T_C_ exclusion and T_H_1_Reg_ enrichment. These data support and add to recent evidence suggesting that the dominant role for PDL1 in CHL is to modulate the tumor-protective immune tumor microenvironment rather than maintaining an exhausted T-cell infiltrate.^11,12^

Reversal of exhaustion is the commonly accepted mechanism of action of PD1 inhibitors in both solid and hematologic malignancies. However, recent research on CHL has shown that expansion of novel T-cell clones, increased baseline T_H_ receptor diversity and retained CHL MHC II expression but not increased T_C_ signatures or reinvigoration of existing (putatively exhausted) clones are associated with PD1 inhibitor response.^11,12^ This raises questions as to whether reversal of exhaustion is the dominant mechanism of action of PD1 inhibitors in CHL. Exhaustion is assumed to be present because of high PDL1 expression and sensitivity to PD1 inhibition; however, this is largely extrapolated from solid tumors and chronic viral infection in which T_C_ exhaustion is well characterized.^3^ In contrast to solid tumors, CHL cells are derived from professional antigen-presenting cells within a T_H_-dominated immune environment and frequently lose MHC-I expression. It is therefore plausible that the interactions with immune cells would be different from the classical tumor-exhausted effector relationship. Evidence for exhaustion in CHL is limited. PD1 expression has been noted to be lower in CHL than in reactive tissue and similar to that in diffuse large B-cell lymphoma (which is much less responsive to PD1 inhibitors).^9,32^ Deep phenotyping has detected a terminally differentiated T_H_ effector subset with T_H_1-like characteristics.^33^ No study has demonstrated the presence of functional exhaustion in CHL.

Our data inform and expand upon those in the literature and on previous work from our group in a new cohort, providing a more detailed functional assessment than any other to date but again finding no evidence of exhaustion above that seen in reactive tissue. Importantly, we also demonstrated that the effects of PDL1 both between CHL cases and spatially within individual tumors are not related to exhaustion signatures. This lack of relationship highlights that PDL1 is not a reliable surrogate for exhaustion, and the conclusion that PDL1 may therefore play roles beyond maintaining exhaustion perhaps explains why PDL1 has consistently been reported to predict PD1 inhibitor response in CHL while PD1 never has.^6^ We went on to identify a consistent link between PDL1 expression and T_H_1_Reg_ enrichment, supporting our hypothesis that a dominant role of PDL1 within the tumor microenvironment is to sculpt the immune infiltrate by maintaining a regulatory T_H_ environment.

The link between PDL1-PD1 signaling and the development of T_H_1_Reg_ is supported by a body of literature. Activated T cells upregulate PD1 and become highly sensitive to PDL1-PD1 signaling at even low levels of PD1 expression.^23^ Engagement of PD1 influences T-cell phenotype at multiple stages of differentiation. During activation PD1 engagement by PDL1 promotes differentiation of naïve T_H_ cells towards T_Reg_ and limits the development of effectors such as T_H_1.^24,25,34–36^ PDL1 promotes plasticity of fully differentiated T_H_1 towards T_Reg_.^27,28^ PDL1 also stabilizes the conversion of inducible T_Reg_ to T_H_1_Reg_.^29,30,37,38^ These mechanisms point to a common concept – that of PDL1 expression shifting T-cell development away from anti-tumor effectors including T_H_1 and promoting development of tumor-protective regulatory cells including T_H_1_Reg_. This pattern is borne out by our data. The combined association we observed between PDL1 expression, retained CHL MHC-II expression and T_H_1_Reg_ enrichment suggests this may at least in part be related to CHL antigen presentation and differentiation of naïve T_H_ to T_H_1_Reg_ and it is notable that CHL MHC-II expression is also predictive of response to PD1 inhibitors.^6^ Future work will examine whether the link between PDL1, CHL MHC-II and T_H_1_Reg_ is via a direct or indirect effect and make comparisons to other PD1-responsive tumor types. The data cited support the possibility of a direct mechanistic link but further evidence is required to demonstrate this. It is noteworthy that while a strong correlation is seen, PD1 inhibitor response is not perfectly predicted by PDL1 or MHC-II expression.^6,20^ It is likely that PD1 inhibition acts via multiple pathways, beyond both exhaustion and the T_H_ compartment, and other mechanisms including invigoration of innate immunity or B-cell compartments may play important roles.^11,12,15^ Our findings also provide a perspective on combination therapies such as PD1 and LAG3 co-inhibition, suggesting that rather than both focusing on exhausted cells these approaches may influence two prominent elements of the protective regulatory microenvironment (FOXP3^+^TBET^+^ T _H_1_Reg_ and FOXP3^–^LAG3^+^ T _R_1). This may be particularly appealing given that while both are present irrespective of CHL MHC-II status, T_H_1_Reg_ are enriched in MHC-II-positive CHL, while T_R_1 are enriched in and mechanistically linked to CHL MHC-II loss.^19^

In conclusion, our data build on and enhance the current understanding of the roles of PDL1 within the CHL micro-environment. We provide phenotyping and functional evidence suggesting a limited role for exhaustion in the CHL microenvironment and identify a broader role for PDL1 within the microenvironment with links to MHC-II expression, T_H_ recruitment and the regulatory cell infiltrate. These findings are informative for our understanding of CHL cell survival within the immune microenvironment and together with recent publications reinforce the concept that the reversal of exhaustion may be too narrow a lens with which to view the activity of the PD1-PDL1 axis in CHL.

## Supplementary Material

Supplementary Appendix**Figure:**
