# Supplementary material for: PDL1 shapes the classical Hodgkin lymphoma microenvironment without inducing T-cell exhaustion
Source: Haematologica. 2022 Jul 14;108(4):1068–82. doi: 10.3324/haematol.2022.280014 (PMC10071123; doi:10.3324/haematol.2022.280014)
Supplement: Supplementary Appendix [file 2021_280014_TAYLOR_SUPPL.pdf]

PDL1 shapes the classical Hodgkin lymphoma microenvironment without inducing T-cell exhaustion

Joseph G. Taylor,[1,2] Edward Truelove,[1,2] Andrew Clear,[1] Maria Calaminici[1,2] and John G. Gribben[1,2]

[1]Centre for Haemato-Oncology, Barts Cancer Institute, Queen Mary University of London, and [2]Barts NHS Trust, St Bartholomew's Hospital, London, UK

Correspondence: J.G. Gribben
j.gribben@qmul.ac.uk

Received: September 25, 2021.
Accepted: June 9, 2022.
Early view: July 14, 2022.

https://doi.org/10.3324/haematol.2021.280014

Published under a CC BY license

Supplementary Tables

Antibodies for Immunohistochemistry

TARGET	CLONE	VENDOR	CATALOGUE NO.	DILUTION	STRIP VALIDATION
**CD68**	KP1	Dako	GA609	1:8000	Pass
**CD3**	LN10	Leica	CD3-565-L-CE	1:500	Pass
**CD4**	4B12	Leica	368-L-CE	1:500	Pass
**CD8**	C8/144B	Dako	M7103	1:400	Fail
**CD30**	BerH2	Dako	M0751	1:200	Pass
**EOMES**	R poly	Atlas	HPA028896	1:200	Pass
**FOXP3**	263A/E7	Abcam	ab20034	1:500	Pass
**GATA3**	D13C9	Cell Signaling	5852	1:50	Unassessed
**LAG3**	R poly	Atlas	HPA013967	1:200	Pass
**LMP1**	CS.1-4	Dako	M0897	1:12000	Fail
**MHC-II**	CR3/43	Dako	M0775	1:500	Pass
**PD1**	NAT105	Abcam	ab52587	1:25-50	Pass
**PD1**	EH33	Cell Signaling	43248	1:50	Pass
**PD1**	D4W2J	Cell Signaling	86163	1:50	Pass
**PDL1**	E1L3N	Cell Signaling	13684	1:200	Pass
**TBET**	4B10	Santa Cruz	sc21749	1:500	Pass
**TIM3**	G poly	R&D systems	AF2365	1:200	Pass
**RORγT**	6F3.1	Merck	MABF81	1:1000	Pass

R poly = rabbit polyclonal, G poly = goat polyclonal

Antibodies for Imaging Mass Cytometry

TARGET	CLONE	VENDOR	CATALOGUE NO.	DILUTION	METAL TAG
**ALPHA SMA**	1A4	Fluidigm	3141017D	1:200	141Pr
**HLA-DR**	LN3	Biolegend	327002	1:50	142Nd
**CD34**	ICO115	Cell Signaling	3569BF	1:25	143Nd
**CD14**	EPR3653	Fluidigm	3144025D	1:100	144Nd

**TBET**	D6N8B	Fluidigm	3145015D	1:50	145Nd
**CD16**	EPR16784	Fluidigm	3146020D	1:50	146Nd
**CD163**	EDHu-1	Fluidigm	3147021D	1:100	147Sm
**CD30**	E4L4I	Cell Signaling	54535BF	1:40	148Nd
**CD10**	Polyclonal	R&D Systems	AF1182	1:50	150Nd
**CD31**	EPR3094	Fluidigm	3151025D	1:100	151Eu
**CD45**	CD45-2B11	Fluidigm	3152018D	1:100	152Sm
**LAG3**	D2G40	Fluidigm	3153028D	1:50	153Eu
**CD11C**	D3V1E	Cell Signaling	45581BF	1:50	154Sm
**FOXP3**	236A/E7	Fluidigm	3155016D	1:50	155Gd
**CD4**	EPR6855	Fluidigm	3156033D	1:200	156Gd
**IRF4**	Polyclonal	R&D Systems	AF5525	1:62.5	158Gd
**CD68**	KP1	Fluidigm	3159035D	1:50	159Tb
**CD20**	H1	Fluidigm	3161029D	1:400	161Dy
**CD8A**	C8/144B	Fluidigm	3162034D	1:100	162Dy
**TIM3**	D5D5R	Cell Signaling	45208BF	1:25	164Dy
**PDL1**	E1L3N	Cell Signaling	13684BF	1:50	165Ho
**B7H4**	H74	Fluidigm	3166030D	1:50	166Er
**GRB**	EPR20129-217	Fluidigm	3167021D	1:100	167Er
**KI67**	B56	Fluidigm	3168022D	1:50	168Er
**COL1**	Polyclonal	Fluidigm	3169023D	1:300	169Tm
**CD3**	Polyclonal	Fluidigm	3170019D	1:100	170Er
**CX3CR1**	8E10.D9	Biolegend	824001	1:25	171Yb
**PD1**	EH12.2H7	Cell Signaling	86163BF	1:33	172Yb
**CD45RA**	HI100	Biolegend	304102	1:66	173Yb
**CD206**	E2L9N	Cell Signaling	91992BF	1:33	175Lu
**HISTONE H3**	D1H2	Fluidigm	3176023D	1:300	176Yb
**DNA1**	DNA Intercalator	Fluidigm	201192B	1:1000	191Ir
**DNA2**	DNA Intercalator	Fluidigm	201192B	1:1000	193Ir

Reagents for Immunohistochemistry

REAGENT	MANUFACTURER	ITEM CODE	DILUTION
**ANTIGEN UNMASKING SOLUTION**	Vector	H3300	1:100
**ANTIBODY DILUENT**	Zytomed	ZUC025-500	1:1
**SUPERSENSITIVE™ POLYMER-HRP**	Biogenex	QD440-XAKE	neat
**DAB SUBSTRATE**	Biogenex	QD440-XAKE	As per kit
**VIP PEROXIDASE (HRP)**	Vector	SK-4600	As per kit
**HAEMOTOXYLIN SOLUTION GILL II**	Sigma	GH216	
**SCOTT'S SOLUTION**	Sigma	S5134-6X	1:10
**DPX MOUNTANT**	Sigma	06522	neat

Antibodies for Flow Cytometry

ANTIGEN	VENDOR	FLUORO	CAT #	CLONE	DIL/TEST
**DAPI**	BD Bio	*live/dead*	564907	-	1:2000
**CFSE**	Thermo	*proliferation*	C34554	-	
**CD3**	Biolegend	BV421	300434	UCHT1	2.5 μl
**CD3**	BD Bio	FITC	561806	UCHT1	2.5 μl
**CD3**	Biolegend	PercP-Cy5.5	300430	UCHT1	2.5 μl
**CD3**	Biolegend	*functional*	300438	UCHT1	10 μg/ml
**CD4**	Biolegend	APC-Fire 750	344638	SK3	2.5 μl
**CD4**	Biolegend	APC-Cy7	300518	RPA-T4	1 μl
**CD4**	Biolegend	PE-Cy7	317414	OKT4	1 μl
**CD25**	Thermo	APC-efl780	47-0251-82	CD25-4E3	1μl
**CD28**	Biolegend	*functional*	302934	CD28.2	1μg/ml
**CD127**	Biolegend	FITC	351312	A019D5	1μl
**CD183**	BD	PE-Cy5	551128	1C6/CXCR3	2μl
**CD194**	Biolegend	BV604	359418	L291H4	1μl
**CCR10**	BD	PercP-Cy5.5	564772	1B5	1μl
**FOXP3**	Biolegend	PE-Daz 594	320126	206D	5μl
**IFNγ**	eBioscience	APC	17-7319-41	4S.B3	2.5μl
**IL2**	BD Bio	PE	554566	MQ1-17H12	2.5μl

**KI67**	Biolegend	BV711	350516	Ki-67	2.5μl
**PD1**	BD Bio	BB515	564494	EH12.1	2.5μl
**PD1**	eBioscience	PE	12-2799-42	eBioJ105	2.5μl
**TBET**	BD Bio	BV711	563320	O4-46	5μl
**ZY**	Biolegend	*live/dead*	423104	-	1:1000

Reagents for Flow Cytometry

REAGENT	DETAILS
**BD BRILLIANT STAIN BUFFER**	BD Bio (cat. 563794), 50ul/test when 2 Brilliant™ antibodies used in same cocktail
**CELL STIMULATION COCKTAIL**	eBioscience (cat. 00-4970-93), 1:500
**IC FIX & PERM BUFFER SET**	eBioscience (cat. 88-8824-00)<br>(contains IC fix buffer, Perm wash buffer)
**PROTEIN TRANSPORT INHIBITORS**	eBioscience (cat. 00-4980-03), 1:500
**TRANSCRIPTION FACTOR BUFFER SET**	BD Bioscience (cat. 562574)<br>(contains Fix/Perm solution, Perm/Wash buffer)
**LYMPHOPREP**	Stemcell Technologies (Cat# 07801)
**RED CELL LYSIS BUFFER**	Biolegend (Cat# 420301)
**CD14 MICROBEADS HUMAN**	Miltenyi (cat. 130-050-201)
**NAÏVE CD4 ISOLATION KIT**	Stemcell EasySep (cat. 17555).

Full Immunohistochemistry protocol:

Sections were oven-dried at 60°C overnight before incubations in xylene (2x 2 minute), industrial methylated spirits (IMS) (1x 2 minute), IMS with 2% hydrogen peroxide (2x 5 minute), IMS (1x 2 minute) before submerging in running tap water. Heat-induced epitope retrieval was performed for 10 minutes at pressure in a pressure cooker in citric acid based antigen unmasking solution before cooling in running tap water and transfer to tris-buffered saline with tween (TBS-T). Antibody

incubation was for forty minutes at room temperature (RT) before detection with SuperSensitive™ Polymer-HRP Kit (20 minutes in Super-Enhancer, 30 minutes in SS-Label separated with TBS-T washes) before detection with DAB or VIP chromogen for 10 minutes. After detection counterstain was for 5 minutes in Gills II Haematoxylin, transfer to running tap water (2 minutes), 5 dips in acid-alcohol, running tap water (2 minutes) then bluing in Scott's solution (3 minutes), dehydration with changes of IMS (3x 2 minute) and clearing with xylene (3x 2 minute). Finally, DPX mounting and scanning. Coverslip removal post scanning by soaking in xylene.

Multiplex Immunohistochemistry protocol:

Multiplex IHC (mIHC) was performed using the same protocol as above. VIP chromogen and the associated primary antibody is removed by Heat Induced Epitope Retrieval, whereas DAB chromogen is heat stable and cannot be removed. For multiplex staining the standard IHC protocol was completed in full before scanning and repeating in full selecting VIP or DAB chromogen depending on whether signal was to be retained or stripped (Figure S1). For all mIHC panels validation was performed in tonsil with both stripped and fresh stained tonsil sections assessed to monitor for excessive background or signal loss with serial stripping iterations. Most antibodies strip completely. For validation the antibody was stained as per the standard IHC protocol with VIP chromogen, scanned, then the protocol was repeated without adding further antibody but completing all antibody detection steps and without counterstain to assess for residual antibody signal (Figure S2). Results of validation reported in the Antibodies for Immunohistochemistry table (Supplementary methods, above). Antibodies that failed stripping validation were placed last in stripping panels.

Full Imaging Mass Cytometry protocol

In-house metal conjugation of antibodies was with Maxpar X8 labelling kits (Fluidigm) according to manufacturer's instruction. Antibody recovery was assessed with Nanodrop (Thermo Fisher) and conjugated antibodies were stored at 4°C in Candor Antibody stabiliser (Candor Bioscience) with 0.05% sodium azide. Antigen retrieval was performed as per IHC staining protocol up to and including pressure cooker before washing for 10 minutes in distilled water, rinsing twice in TBS-T,

washing twice in PBS and blocking with SuperBlock at room temperature for 45 minutes. Antibody master-mix was prepared in antibody diluent in a 0.1μm spin-filter and centrifuged at 12000 rcf for 1 minute to remove antibody aggregates. Slides were then incubated with 200μl of master-mix in a hydration chamber at 4°C overnight. Slides were washed twice in TBS-T and twice with PBS, before nuclear staining with iridium intercalator in PBS in a hydration chamber at room temperature for 30 minutes. Slides were then washed twice in distilled water and air died overnight. Prior to acquisition, the Hyperion imaging mass cytometry system was tuned with a 3-element tuning slide (Fluidigm) for daily quality control (QC) as per manufacturer's protocol. TMA cores were ablated at 200Hz. Stain quality was compared to IHC (Figure S3). MCD files were converted to tiff image stacks and segmented to extract single cell data for further analysis (https://github.com/BodenmillerGroup/ImcSegmentationPipeline) as previously described.[1,2] Briefly, random image crops were generated, scaled-up x 2 and pixels manually classified as nuclear, membrane/cytoplasmic or background using a combination of CellProfiler (version 4.2.1) and Ilastik (version 1.4.0b15). The trained classifier was used to generate probability masks from which single cell data was extracted in R.

Flow cytometry Protocol:

Flow cytometry surface staining was performed for 20 minutes. Cytokine staining used the IC Fix & Perm buffer set with 40-minute fixation and 40-minute intracellular staining steps. Transcription factor staining used the Transcription factor buffer set with 40-minute fixation, 30-minute Perm/Wash incubation and 40-minute intracellular staining steps. All incubations were performed at RT. Viability stains were included in all cocktails.

R Packages:

broom (v0.5.6), conflicted (v1.0.4), cowplot (v1.0.0), corrr (v0.4.2), extrafont (v0.17), formattable (v0.2.1), future (v1.17.0), ggpubr (v0.3.0), ggsignif (v0.6.0), ggthemes (v4.2.0), here (v0.1), pacman (v0.5.1), psych (v2.1.6), readxl (v1.3.1), rstatix (v0.6.0), scales (v1.1.1), spatstat (v1.63-3), tidyverse (v1.3.0), viridis (v0.5.1), wrapr (v2.0.0), XML (v3.99-0.3),

Supplementary Figures

Figure S1: Serial Stripping of Antibodies for Virtual Multiplex IHC

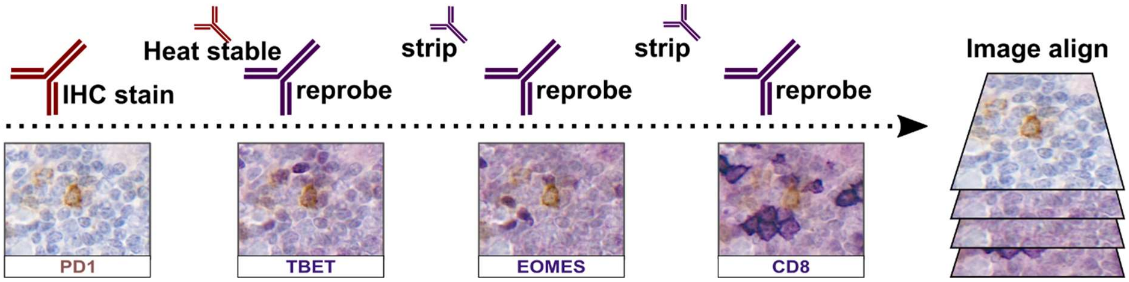

Legend: Antibodies and serially stained, scanned before stripping and restaining. Images are virtually aligned for multiplex analysis. DAB (brown) chromogen is selected when stain is to be retained due to being heat stable so not removed by heat induced epitope retrieval. VIP (purple) chromogen is selected when stain is to be removed due to being heat unstable so both the chromogen and associated primary antibody are stripped by heat induced epitope retrieval.

Figure S2: Antibody Validation for Inclusion in Multiplex IHC Stripping Panels

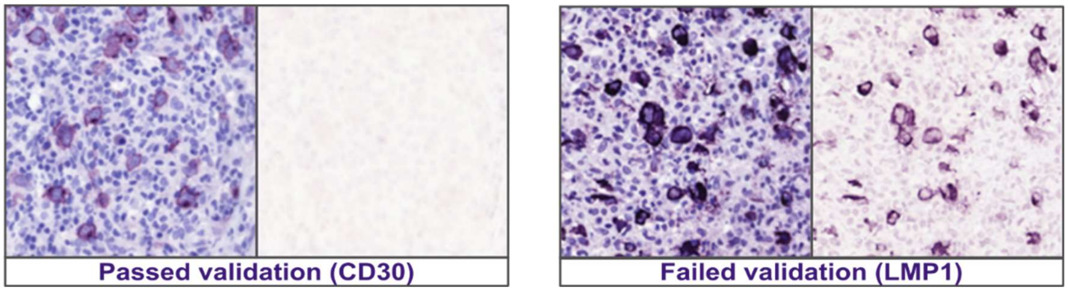

Legend: Antibodies are validated for stripping by assessing residual signal on restaining. Antibodies are stained using the standard IHC protocol (methods) and VIP (purple) chromogen. Staining process is repeated in full but omitting the addition of a second antibody or counterstain. Antibody is removed by heat induced epitope retrieval step. Antibodies are validated for stripping when no residual signal is seen (CD30, left). In some cases, residual signal remains (LMP1, right). These antibodies are placed last in a staining panel to avoid compromising subsequent stains within the panel. Conventional IHC controls were stained in parallel to stripping panels to assess signal loss and background due to the stripping process. Staining performed using DAKO Autostainer.

Figure S3: Immunohistochemistry vs. Imaging Mass Cytometry validation

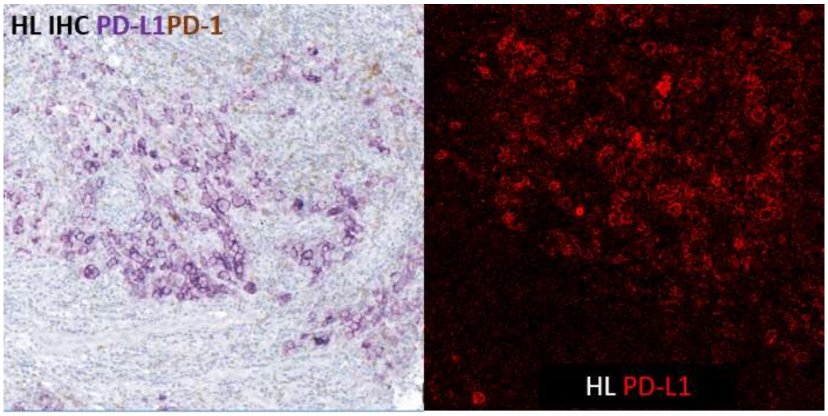

Legend: Comparison of stain quality between IHC and IMC in paired section.

Figure S4: PD1 IHC Antibody Clone Validation

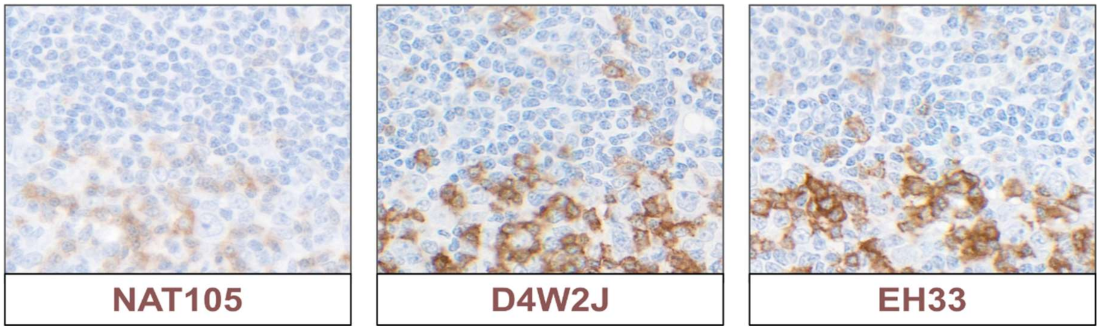

Legend**:** PD1 clones vary in sensitivity. Superior identification of weak PD1 expression is seen with anti-PD1 clones D4W2J and EH33 as compared to NAT105. IHC in serial tonsil sections. NAT105 (1:25 dilution), D4W2J (1:50 dilution), EH33 (1:50 dilution). Clone D4W2J was used for analysis in this study due to its high sensitivity. Results were validated with clone NAT105 which is approved for diagnostic use.

Figure S5: The CHL Single Cell Suspension Cohort was Similar to the Wider IHC Cohort

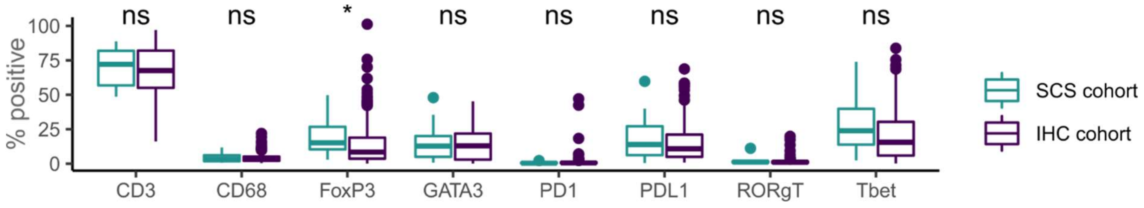

Legend: The Single Cell Suspension (functional) cohort is representative of the IHC cohort. Paired Formalin-Fixed Paraffin-Embedded presenting biopsies of cases with single cell suspension samples used for functional testing were compared by IHC to the wider IHC cohort. The functional cohort was similar in terms of CD3, PD1, PDL1, CD68, TBET, GATA3 and RORγT expression. Significantly more FOXP3 (associated with T_{Reg}) was detected in the functional cohort as compared to the wider IHC cohort (comparison by Mann Whitney, p = 0.044).

Figure S6: CHL Proliferation and Cytokine-competence Varies by EBV Status

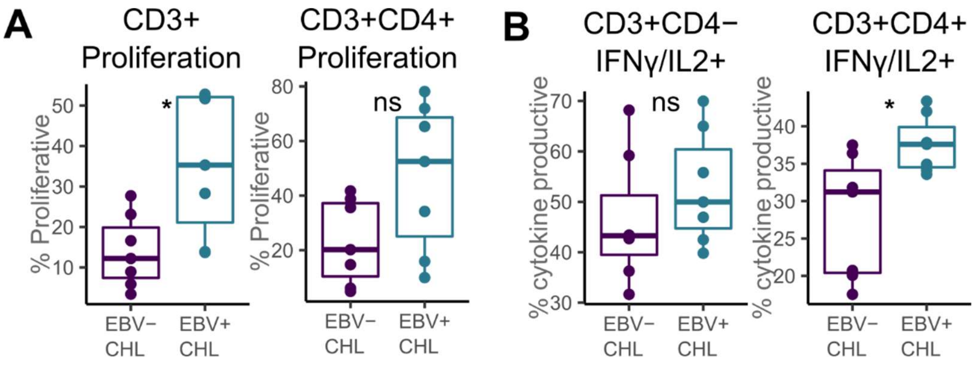

Legend: EBV⁺CHL is associated with higher T cell proliferation and higher T_H cytokine responsiveness than EBV⁻CHL. EBV⁺CHL T cells were more proliferative than EBV⁻CHL T cells to CD3/CD28 stimulation in vitro by CFSE assay, Mann Whitney, EBV^- n = 8, EBV^+ n = 7, p = 0.017). Similarly, a greater fraction of EBV^+CHL T_H cells produced IFNγ or IL2 to PMA/Ionomycin stimulation in vitro than EBV^-CHL T cell. (Mann Whitney, EBV^- n = 8, EBV^+ n = 7, p = 0.017)

Figure S7: PD1 does not associate with CHL MHC II expression

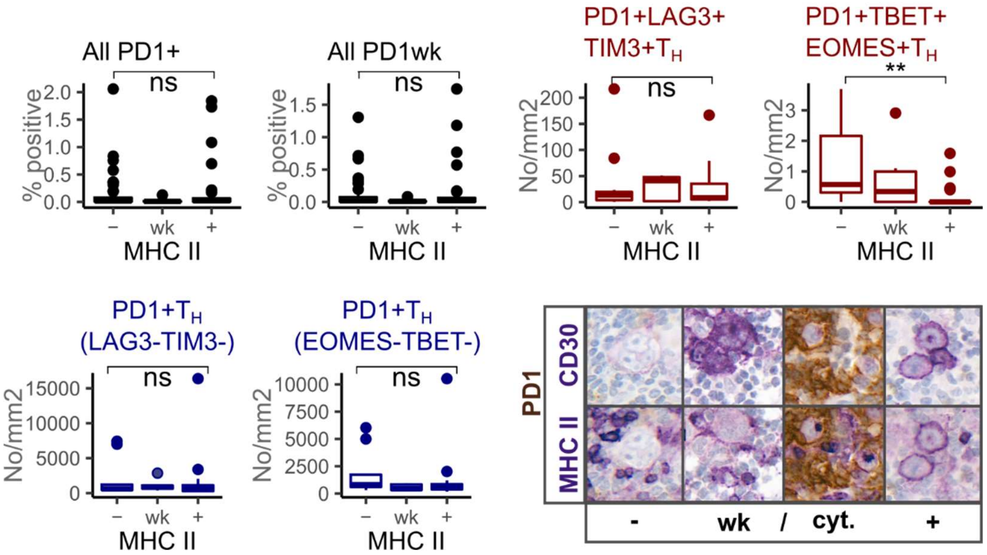

Legend: PD1 is not associated with CHL MHC-II expression. CHL cases were manually scored as MHC-II positive, MHC-II weak or cytoplasmic or MHC-II negative (image: Top - CD30 purple, PD1 brown. Bottom – MHC-II purple, PD1 brown). No significant association was detected between either PD1 or $PD1^{weak}$ to MHC-II overall (MHC-II- n = 47, MHC-II+ n = 54) and a significant inverse relationship was detected between CHL MHC=II and $PD1^{+}TBET^{+}EOMES^{+}T_{H}$ frequency (MHC-II- n = 12, MHC-II+ n = 17, p = 0.0024). Statistical comparison by Mann Whitney between positive and negative group because weak category is of indeterminate significance. MHC-II staining was scored manually.
